# Whole-Body ^12^C Irradiation Transiently Decreases Mouse Hippocampal Dentate Gyrus Proliferation and Immature Neuron Number, but Does Not Change New Neuron Survival Rate

**DOI:** 10.3390/ijms19103078

**Published:** 2018-10-09

**Authors:** Giulia Zanni, Hannah M. Deutsch, Phillip D. Rivera, Hung-Ying Shih, Junie A. LeBlanc, Wellington Z. Amaral, Melanie J. Lucero, Rachel L. Redfield, Matthew J. DeSalle, Benjamin P. C. Chen, Cody W. Whoolery, Ryan P. Reynolds, Sanghee Yun, Amelia J. Eisch

**Affiliations:** 1 Department of Anesthesiology and Critical Care Medicine, Children’s Hospital of Philadelphia, Philadelphia, PA 19104, USA; giulia.zanni85@gmail.com (G.Z.); hdeu@sas.upenn.edu (H.M.D.); desallem7@gmail.com (M.J.D.); ryan.p.reynolds07@gmail.com (R.P.R.); YUNS@email.chop.edu (S.Y.); 2 Biological Basis of Behavior Program, University of Pennsylvania, Philadelphia, PA 19104-6270, USA; 3 Department of Psychiatry, UT Southwestern Medical Center, Dallas, TX 75390, USA; phrivera@gmail.com (P.D.R.); junie_leblanc@hotmail.com (J.A.L.); wamaral@wisc.edu (W.Z.A.); Mjlucero1@gmail.com (M.J.L.); rachelredfield22@gmail.com (R.L.R.); cody.whoolery@gmail.com (C.W.W.); 4 Department of Radiation Oncology, UT Southwestern Medical Center, Dallas, TX 75390, USA; Eric.Shih@UTSouthwestern.edu (H.-Y.S.); Benjamin.Chen@UTSouthwestern.edu (B.P.C.C.); 5 Perelman School of Medicine, University of Pennsylvania, Philadelphia, PA 19104, USA; 6 Mahoney Institute for Neurosciences, Perelman School of Medicine, University of Pennsylvania, Philadelphia, PA 19104, USA

**Keywords:** space radiation, galactic cosmic radiation, carbon, hippocampus, neurogenesis, subgranular zone

## Abstract

High-charge and -energy (HZE) particles comprise space radiation and they pose a challenge to astronauts on deep space missions. While exposure to most HZE particles decreases neurogenesis in the hippocampus—a brain structure important in memory—prior work suggests that ^12^C does not. However, much about ^12^C’s influence on neurogenesis remains unknown, including the time course of its impact on neurogenesis. To address this knowledge gap, male mice (9–11 weeks of age) were exposed to whole-body ^12^C irradiation 100 cGy (IRR; 1000 MeV/n; 8 kEV/µm) or Sham treatment. To birthdate dividing cells, mice received BrdU i.p. 22 h post-irradiation and brains were harvested 2 h (Short-Term) or three months (Long-Term) later for stereological analysis indices of dentate gyrus neurogenesis. For the Short-Term time point, IRR mice had fewer Ki67, BrdU, and doublecortin (DCX) immunoreactive (+) cells versus Sham mice, indicating decreased proliferation (Ki67, BrdU) and immature neurons (DCX). For the Long-Term time point, IRR and Sham mice had similar Ki67+ and DCX+ cell numbers, suggesting restoration of proliferation and immature neurons 3 months post-^12^C irradiation. IRR mice had fewer surviving BrdU+ cells versus Sham mice, suggesting decreased cell survival, but there was no difference in BrdU+ cell survival rate when compared within treatment and across time point. These data underscore the ability of neurogenesis in the mouse brain to recover from the detrimental effect of ^12^C exposure.

## 1. Introduction

Over fifty years ago, Altman and Das showed new neurons are generated in the adult mammalian hippocampal dentate gyrus (DG) [1,2,3], thus disproving the long-standing belief that neurogenesis ceases at birth. We now know a great deal about the function and regulation of adult DG neurogenesis, including its role in memory and mood [4,5,6,7,8,9], its regulation by oxidative stress [10,11,12,13,14,15], and its exceptional sensitivity to types of radiation [16,17,18,19,20]. Of note, the high-charge and high-energy (HZE) particles—such as ^56^Fe and ^28^Si—that comprise cosmic radiation have been extensively studied for their ability to impact DG neurogenesis in laboratory animals [21], with most work reporting a negative impact of space radiation on indices of neurogenesis. Functionally, HZE exposure has been linked to accelerated aging, anxiety-like symptoms, spatial learning and memory deficits, and decreased operant responses in laboratory animals [22,23,24,25,26,27,28], all of which might be related to decreased DG neurogenesis. As HZE particle exposure is an unavoidable aspect of deep space missions [29,30,31,32,33], it is important to gain a complete understanding of how exposure to distinct HZE particles influences DG neurogenesis.

When compared to the many HZE particles that comprise space radiation, carbon (^12^C) has received far less attention in regard to its impact on DG neurogenesis. This is somewhat surprising given that ^12^C irradiation has emerged as a potential cancer treatment [34,35]. Specifically, ^12^C is a good candidate to replace X-irradiation as the ability to target this heavy radiation beam enables sparing of the normal tissue surrounding a tumor [36,37,38]. Even though heavy particle therapy for cancer treatment is typically provided in a focal beam and HZE particle work relevant to space radiation is typically provided as whole-body irradiation, the underlying mechanisms of how ^12^C disrupts brain and behavior in the context of space travel is likely to also be relevant to heavy particle therapy.

While it is widely accepted that HZE irradiation decreases neurogenesis, only one study has examined the impact of ^12^C on DG neurogenesis [39]. The authors studied the indices of neurogenesis (with a quantitative assessment of cells immunoreactive [+] for Ki67 and doublecortin [DCX], and a qualitative assessment of cells immunoreactive for the thymidine analog bromodeoxyuridine [BrdU]) and a neuronal nuclear protein [NeuN]) 9-months [mon] post-^12^C irradiation (100–300 cGy, 290 MeV/n, and linear energy transfer [LET] 13 keV/µm). Despite the authors’ conclusion that the “changes [in neurogenesis] are not only persistent but may worsen with time” [39], their statistical analyses do not indicate decreased neurogenesis. Even though HZE particle-induced changes in DG neurogenesis are known to be dynamic [40,41], the authors examined only a single time point post-^12^C irradiation (9 mon). Thus, the conclusion drawn by Rola et al., 2005 that these changes are “persistent and progressive” [39] merits revisitation with the inclusion of an earlier time point. In addition, the authors sampled indices of neurogenesis at one level of the hippocampus (rostral/mid hippocampus). As the hippocampus is a large structure driving diverse functions along its longitudinal axis [4,42,43,44,45,46], stereological assessment might reveal previously-overlooked changes in neurogenesis, particularly in the caudal or posterior hippocampus. Finally, as ^12^C exposure may result in more cognitive deficits than equivalent doses of larger particles, such as ^28^Si and ^48^Ti [47], studies that more closely examine ^12^C impact on DG neurogenesis are warranted.

To address these knowledge gaps, here we define how whole-body ^12^C irradiation affects DG hippocampal neurogenesis in male mice. A major consideration of our work was to facilitate comparison with how other space radiation particles influence brain and behavior (^1^H [22,23,48,49,50,51,52,53,54,55,56,57,58,59], ^4^He [60,61,62,63], ^12^C [22,39,47,64,65], ^16^O [22,47,54,60,66,67,68,69,70,71,72,73,74], ^28^Si [22,39,41,47,51,56,58,75,76,77,78], ^48^Ti [22,47,58,60,68,69,79], and ^56^Fe [22,23,24,25,28,39,40,48,51,55,56,71,80,81,82,83,84,85,86,87,88,89,90,91,92,93,94,95,96,97,98,99,100,101,102,103,104,105,106,107,108,109,110,111,112,113,114,115,116,117,118,119,120,121]). To this end, we age- and sex-matched our subjects with the bulk of existing literature, choosing to use young adult male mice. We mimicked space radiation using ground-based accelerators at NASA’s Space Radiation Laboratory (NSRL) located at Brookhaven National Laboratories (BNL). Consistent with prior literature using other particles, mice received a single exposure. To build on prior work with ^12^C, we examined neurogenesis at both short (24 h [h]) and long (three mon) time points after irradiation. We find whole-body ^12^C irradiation transiently decreases indices of proliferation and immature neurons in the mouse DG, but it does not change new neuron survival rate. These data underscore the ability of neurogenesis in the mouse brain to recover from the detrimental effect of ^12^C exposure.

## 2. Results

### 2.1. ^12^C Irradiation Had No Overt Physiological Influence on IRR Mice Relative to Sham Mice

**Figure 1:**
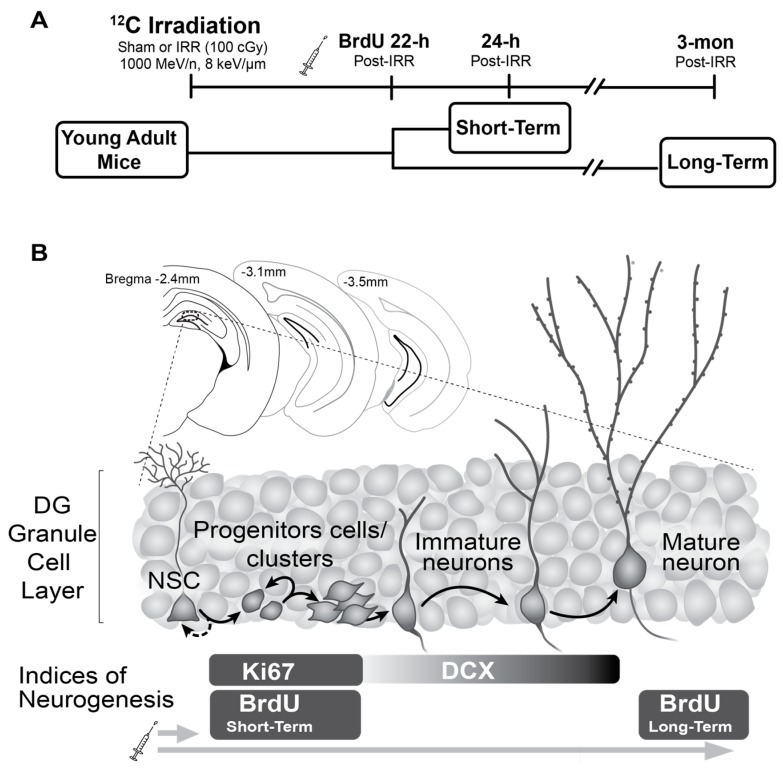
Schematic of experimental design and overview of neurogenesis indices examined. (**A**) Timeline of the experimental design used to investigate the Short- and Long-Term (24 h and 3-mon post-irradiation, respectively) effects of whole-body ^12^C irradiation in young adult mice (9–11 wks at the time of irradiation) on dentate gyrus (DG) neurogenesis. All mice received the mitotic marker bromodeoxyuridine (BrdU) 22-h post-irradiation, with brains collected at a Short-Term or Long-Term time point. (**B**) Graphical representation of the mouse brain region that was the focus of this study. Upper left schematics depict three representative bregma coronal hemisections through the rostral, middle, and caudal hippocampal DG [122]. Main image depicts enlarged DG granule cell layer (GCL). Cells across the stages of DG neurogenesis are shown in the magnified GCL: neural stem cells (NSC), proliferating cells and clusters, immature neurons, and mature DG granule cell neurons. As indicated by the indices of neurogenesis on the bottom, proliferating cells/clusters can be labeled with antibodies against the endogenous proteins Ki67 or the exogenous thymidine analog BrdU (if examined shortly after BrdU injection), immature neurons can be labeled with an antibody against doublecortin (DCX), and surviving granule cell neurons can be labelled with an antibody against BrdU (if examined several weeks after BrdU injection). Figure modified from [41].

Young adult male mice received a single exposure of whole-body ^12^C irradiation (dose 100 cGy; energy 1000 MeV/n; LET 8 kEV/µm) or Sham treatment and brains were collected 24-h or 3-mon post-irradiation (Figure 1A). Similar to other work with whole-body exposure to HZE particles [41], irradiation (IRR), and Sham mice had similar weight before and after irradiation and similar growth rates, and no obvious effects of IRR (such as hair loss, lethargy, or sickness) were seen at any time point.

**Figure 2:**
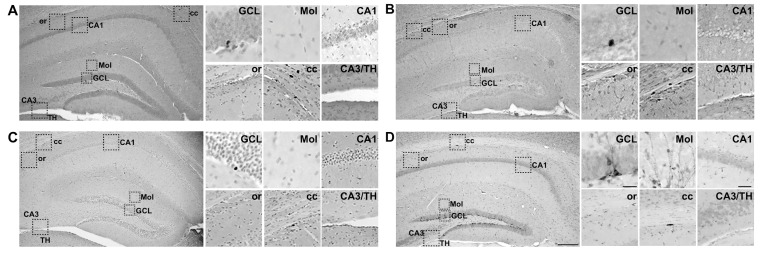
Representative photomicrographs of cells immunoreactive for neurogenesis markers (Ki67, BrdU, and DCX) in the hippocampus. (**A**) Large panel shows Ki67 immunoreactivity in a Sham mouse brain (mouse #14086) at low magnification (100×) collected 24 h post-sham treatment. Regions outlined in large panel (GCL, Mol, CA1, or, cc, CA3/TH) are shown at higher magnification in smaller panels to the right. Ki67+ cells are evident in the Granule Cell Layer (GCL) and corpus callosum (cc), but not in any other visible brain region. (**B**) Large panel shows BrdU immunoreactivity in a Sham mouse brain (mouse #14086) at low magnification (100×) collected 2 h after BrdU injection and 24 h post-sham treatment. Regions outlined in large panel are shown at higher magnification in smaller panels to the right. BrdU+ cells are evident in the GCL and cc, but not in any other brain region. (**C**) Large panel shows BrdU immunoreactivity in a Sham mouse brain (mouse #13851) at low magnification (100×) collected 3 mon after BrdU injection and 3 mon post-sham treatment. Regions outlined in large panel are shown at higher magnification in smaller panels to the right. A distinct BrdU+ cell is evident in the GCL, but not in other brain regions. (**D**) Large panel shows doublecortin (DCX) immunoreactivity in a Sham mouse brain (mouse #13851) at low magnification (100×) collected 3 months after BrdU injection and 3 months’ post-sham treatment. Regions outlined in large panel are shown at higher magnification in smaller panels to the right. DCX+ cells are evident in the GCL and cc, and DCX+ dendritic fibers are evident in the GCL molecular layer (Mol), but immunoreactivity is not seen in any other visible brain region. Scale bar = 200 µm in large panel; 25 µm in GCL and Mol images; 50 µm in CA1, or, cc, and CA3/TH images; applies to A–D. CA1 cornus ammonis 1; CA3 cornus ammonis 3; cc corpus callosum; GCL granule cell layer; Mol molecular layer; or stratum oriens; TH thalamus.

**Figure 3:**
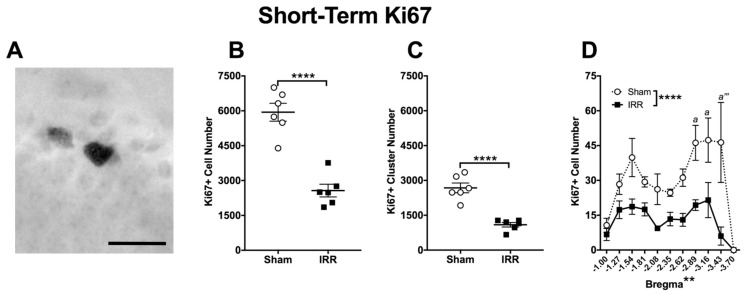
24-h post-irradiation, whole-body exposure to ^12^C irradiation reduced the number of DG GCL Ki67+ cells and clusters relative to Sham treatment. (**A**) Representative photomicrograph of Ki67-immunoreactive (Ki67+) staining in the DG GCL of a Short-Term Sham mouse (mouse #14086; 400×, scale bar = 25 µm). (**B**) An unpaired two-tailed *t*-test revealed fewer Ki67+ cells in the DG GCL 24-h after ^12^C irradiation versus Sham treatment (**** *p* < 0.0001; *N* = 6 Sham, *N* = 6 IRR). (**C**) An unpaired two-tailed *t*-test revealed fewer Ki67+ clusters in the DG GCL 24-h after ^12^C irradiation versus Sham treatment (**** *p* < 0.0001). (**D**) Bregma analysis of Ki67+ cells in the DG GCL through two-way ANOVA (Bregma × Treatment) revealed main effects of Treatment (*F*_1,10_ = 50.7; **** *p* < 0.0001) and Bregma (F_9,90_ = 3.089; ** *p* < 0.01), but no significant interaction (*F*_9,90_ = 1.442; *p* > 0.05). Post-hoc (Sidak’s multiple comparison) test revealed fewer Ki67+ cells at bregma positions −2.89 (*a p* < 0.05), −3.16 (*a p* < 0.05), and −3.43 (*a’’’ p* < 0.0001) between Sham and IRR mice. Error bars ± SEM.

**Figure 4:**
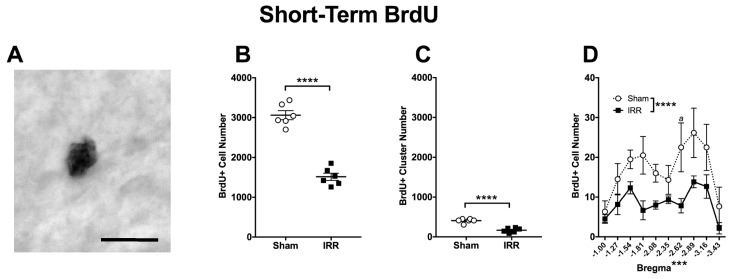
24-h post-irradiation, whole-body exposure to ^12^C irradiation reduced the number of DG GCL BrdU+ cells and clusters relative to Sham treatment. (**A**) Representative photomicrograph of BrdU staining in a Short-Term Sham mouse (mouse #14086; 400×, scale bar = 25 µm). (**B**) An unpaired two-tailed *t*-test revealed fewer BrdU+ cells in the DG GCL 24-h after ^12^C irradiation versus Sham treatment (**** *p* < 0.0001; *N* = 6 Sham, *N* = 6 IRR). (**C**) An unpaired *t*-test revealed fewer BrdU+ clusters in the DG GCL 24-h after ^12^C irradiation versus Sham treatment (**** *p* < 0.0001). (**D**) Bregma analysis of BrdU+ cells in the DG GCL through two-way ANOVA (Bregma × Treatment) revealed main effects of Treatment (*F*_1,10_ = 115.4; **** *p* < 0.0001) and Bregma (*F*_9,90_ = 3.668; *** *p* < 0.001), but no significant interaction (*F*_9,90_ = 0.6751; *p* > 0.05). Post-hoc (Sidak’s multiple comparison) test revealed fewer BrdU+ cells at bregma −2.62 in IRR mice relative to Sham mice (*a p* < 0.05). Error bars ± SEM.

**Figure 5:**
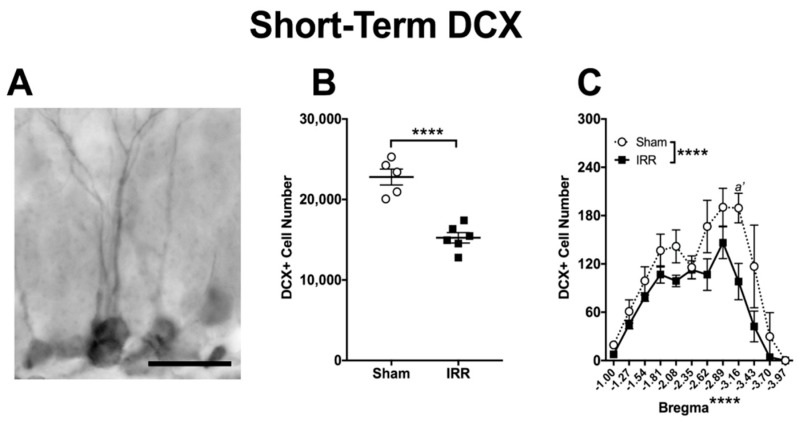
24-h post-irradiation, whole-body exposure to ^12^C irradiation reduced the number of DG GCL DCX+ cells relative to Sham treatment. (**A**) Representative photomicrograph of DCX staining in a Short-Term Sham mouse (mouse #14088; 400×, scale bar = 25 µm). (**B**) An unpaired *t*-test revealed fewer DCX+ cells in IRR versus Sham mice (**** *p* < 0.0001; *N* = 5 Sham, *N* = 6 IRR). (**C**) Bregma analysis of DCX+ cells through two-way repeated measure ANOVA (Bregma × Treatment) revealed main effects of Treatment (*F*_1,9_ = 43.53; **** *p* < 0.0001) and Bregma (*F*_11,99_ = 17.49; **** *p* < 0.0001), but no significant interaction (F_11,99_ = 1.139; *p* > 0.05). Post-hoc (Sidak’s multiple comparison) test revealed fewer DCX+ cells at bregma −3.16 in IRR versus Sham mice (*a’ p* < 0.01). Error bars ± SEM.

**Figure 6:**
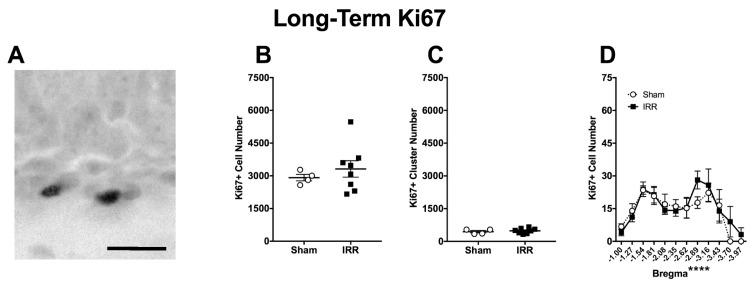
Three-mon post-^12^C irradiation, there was no difference in the number of Ki67+ cells or clusters between IRR and Sham mice. (**A**) Representative photomicrograph of Ki67 staining in a Long-Term Sham mouse (mouse #13850; 400×, scale bar = 25 µm). (**B**) An unpaired *t*-test revealed no difference in the number of Ki67+ cells in the SGZ (*p* > 0.05; *N* = 4 Sham, *N* = 8 IRR). (**C**) An unpaired *t*-test revealed no difference in the number of Ki67+ clusters in the SGZ (*p* > 0.05). (**D**) Bregma analysis of Ki67+ cells in the SGZ through two-way ANOVA (Bregma × Treatment) revealed a main effect of Bregma (*F*_11,110_ = 5.509; **** *p* < 0.0001), but not of Treatment (*F*_1,10_ = 0.2151; *p* > 0.05) and no significant interaction (*F*_11,110_ = 0.4856; *p* > 0.05). Post-hoc (Sidak’s multiple comparison) test revealed no significance at any single bregma. Error bars ± SEM.

**Figure 7:**
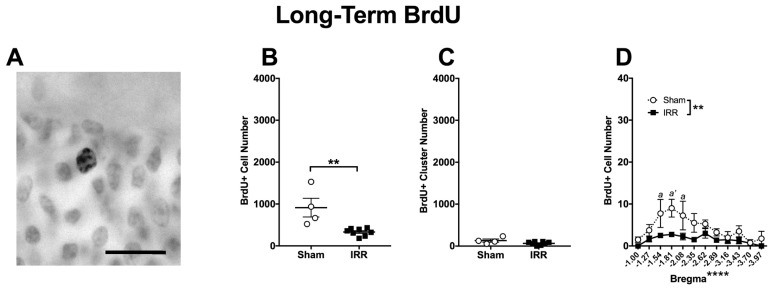
Three-mon post-^12^C irradiation, there were fewer DG GCL BrdU+ cells in IRR mice relative to Sham mice. (**A**) Representative photomicrograph of BrdU staining in a Long-Term Sham mouse (mouse #13853; 400×, scale bar = 25 µm). (**B**) An unpaired *t*-test revealed fewer BrdU+ cells in IRR mice versus Sham mice (** *p* < 0.01; *N* = 4 Sham, *N* = 8 IRR). (**C**) An unpaired *t*-test revealed similar number of BrdU+ clusters in IRR and Sham mice (*p* > 0.05). (**D**) Bregma analysis of BrdU+ cells through two-way ANOVA (Bregma × Treatment) revealed a main effect of Treatment (*F*_1,10_ = 14.31; ** *p* < 0.01) and Bregma (*F*_11,110_ = 5.964; **** *p* < 0.0001), but no significant interaction (*F*_11,110_ = 1.775; *p* > 0.05). Post-hoc (Sidak’s multiple comparison) test revealed fewer BrdU+ cells at bregma positions −1.54 (*a p* < 0.05), −1.81 (*a’ p* < 0.01), and −2.08 (*a p* < 0.05) in IRR mice relative to Sham mice. Error bars ± SEM.

**Figure 8:**
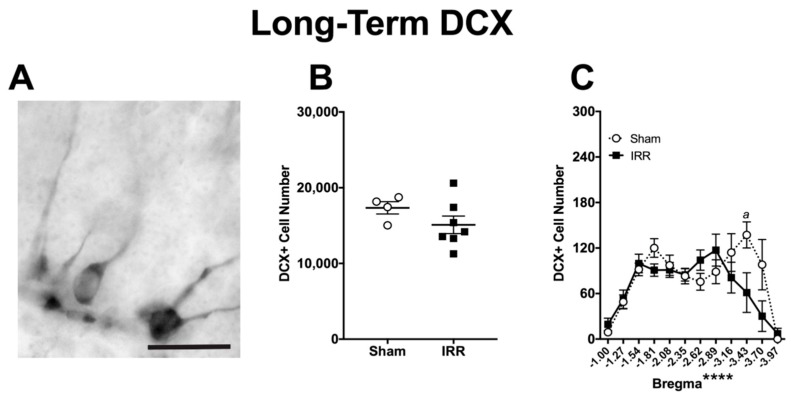
Three-mon post-^12^C irradiation, DCX+ cell number was similar between IRR and Sham mice. (**A**) Representative photomicrograph of DCX staining in a Long-Term Sham mouse (mouse #13851; 400×, scale bar = 25 µm). (**B**) An unpaired *t*-test revealed no difference in DCX+ cells between IRR and Sham mice (*p* > 0.05; *N* = 4 Sham, *N* = 7 IRR). (**C**) Bregma analysis of DCX+ cells through two-way ANOVA (Bregma × Treatment) revealed a main effect of Bregma (*F*_11,99_ = 9.038; **** *p* < 0.0001), but not Treatment (*F*_1,9_ = 1.773; *p* > 0.05) and a significant interaction (*F*_11,99_ = 2.178; *p* < 0.05). Post-hoc (Sidak’s multiple comparison) revealed fewer DCX+ cells at bregma −3.43 (*a p* < 0.05). Error bars ± SEM.

The focus of this study was on hippocampal neurogenesis, neurons that emerge from the progeny of proliferative neural progenitors and mature to become granule cell neurons in the hippocampal DG granule cell layer (GCL). Brains from both the Short-Term group (collected 24-h post-irradiation) and Long-Term group (collected 3-mon post-irradiation; Figure 1A) were processed for immunohistochemistry with antibodies against neurogenesis-relevant antigens Ki67, BrdU, and DCX (Figure 1B). As shown previously [123,124,125], the cells immunoreactive for these antigens were restricted to regions relevant to adult neurogenesis, including the DG GCL (Figure 2). Some immunoreactive cells were also evident in the corpus callosum, as this region is a posterior extension of the anterior neurogenic region the subventricular zone [126,127,128]. As no overt difference in regional distribution of Ki67-immunoreactive (+), BrdU+, or DCX+ cells was observed between the Sham and IRR groups, we used stereology to quantify these cells specifically in the DG GCL of mice in the Short-Term and Long-Term groups to reflect proliferating progenitors, immature granule cell neurons, and mature granule cell neurons (Figure 3, Figure 4, Figure 5, Figure 6, Figure 7 and Figure 8).

### 2.2. 24-h Post-Irradiation, IRR Mice Had 57% Fewer Ki67+ Cells and 59% Fewer Ki67+ Clusters in the DG GCL Relative to Sham Mice

Proliferation was measured at the Short-Term time point through the quantification of Ki67+ cells in the DG GCL (Figure 1B). Ki67+ cells had uneven, dark nuclei (Figure 3A), consistent with previous studies [129,130]. 24-h after ^12^C irradiation, IRR mice had 57% fewer Ki67+ cells versus Sham mice (Figure 3B). As cells and groups of cells—or “clusters”—can be differentially-regulated [123], clusters of Ki67+ cells were also quantified. IRR mice had 59% fewer Ki67+ clusters versus Sham mice (Figure 3C). As the function of the DG varies over the longitudinal axis of the hippocampus (distance from bregma) [44,131,132,133,134], Ki67+ cell number was analyzed over relative distance from bregma. Bregma analysis revealed main effects of Treatment and Bregma, but not a Bregma x Treatment interaction (Figure 3D). Post-hoc analyses revealed three bregma positions (all in posterior DG) where IRR mice had fewer Ki67+ cells relative to Sham (Figure 3D). Thus, at this Short-Term time point, IRR mice had fewer Ki67+ cells and clusters—particularly in the posterior DG—when compared to Sham mice.

### 2.3. 24-h Post-Irradiation, IRR Mice Had 50% Fewer BrdU+ Cells and 59% Fewer BrdU+ Clusters in the DG GCL Relative to Sham Mice

BrdU injected 22-h post-irradiation was used to further explore proliferation in the Short-Term group. As opposed to Ki67, which is endogenously expressed in cells in all stages of the cell cycle, BrdU is an exogenous thymidine analog that incorporates into the cell’s DNA during the S-phase of the cell cycle, thus labeling the nucleus (Figure 4A). In Sham mice, there were ~50% fewer BrdU+ cells in the Short-Term group relative to Ki67+ cells in the Short-Term group (Figure 4B vs. Figure 3B). This comparison of BrdU and Ki67 is consistent with prior estimation of the length of the cell cycle in the mouse DG GCL [129]. 24-h after ^12^C irradiation there were 50% fewer BrdU+ cells and 59% fewer BrdU+ clusters in IRR versus Sham mice (Figure 4B,C). Bregma analysis revealed a main effect of Treatment and Bregma, but not a Bregma × Treatment interaction (Figure 4D). Post-hoc analyses revealed one bregma position in the posterior DG where IRR mice had fewer BrdU+ cells than Sham (Figure 4D). Thus, at this Short-Term time point, IRR mice had fewer BrdU+ cells and clusters—particularly in the posterior DG—when compared to Sham mice.

### 2.4. 24-h Post-Irradiation, IRR Mice Had 33% Fewer DCX+ Cells in the DG GCL Relative to Sham Mice

In addition to proliferating cells, we also measured immature neuron number, as assessed via DCX+ cells. DCX is a microtubule-associated protein expressed in adult-born cells from division into early maturation stages [135], and is even used as a stand-alone index of neurogenesis [135,136,137,138,139,140]. As DCX expression in the DG GCL is associated with a wide range of morphologies reflective of the stage of maturity of the DCX+ cell [141], we quantified DCX+ cells presenting oval- or teardrop-shaped soma with long, thin processes, as well as cells lacking processes (Figure 5A). 24-h after ^12^C irradiation, there were 33% fewer DCX+ cells in IRR versus Sham mice (Figure 5B). Bregma analysis revealed main effects of Treatment and Bregma, but no Bregma × Treatment interaction (Figure 5C). Post-hoc analyses revealed one bregma location in the posterior DG where IRR mice had fewer DCX+ cells relative to Sham mice. Thus, as with Ki67+ cells and BrdU+ cells at this Short-Term time point, IRR mice had fewer DCX+ cells—particularly in the posterior DG—when compared to Sham mice.

### 2.5. Three-mon Post-Irradiation, IRR Mice Had a Similar Amount of Ki67+ Cells and Clusters When Compared to Sham

To complement the data from the Short-Term time point (Figure 3, Figure 4 and Figure 5), proliferation was measured at a Long-Term time point via the quantification of Ki67+ cells (Figure 6). Qualitatively, Ki67+ cells presented a similar morphology in the Short- and Long-Term groups (Figure 3A vs Figure 6A), which is as expected for this endogenous marker of cells in the cell cycle. Quantitatively, in the Sham mice Ki67+ cell numbers were lower in the Long-Term group versus the Short-Term group (Figure 6B vs. Figure 3B, calculations not shown). This is as expected since the proliferation of neural progenitors in the DG decreases with age [142,143,144,145]. However, in the Long-Term group, there was no difference in Ki67+ cell or cluster number between Sham and IRR mice (Figure 6B,C). While Bregma analysis revealed a main effect of Bregma, there was not a main effect of Treatment or a Bregma x Treatment interaction (Figure 6D). Thus, at this Long-Term time point, IRR mice had similar numbers of Ki67+ cells and clusters when compared to Sham mice.

### 2.6. Three-mon Post-Irradiation, IRR Mice Had 64% Fewer BrdU+ Cells, but a Similar Number of Clusters When Compared to Sham Mice

To assess the survival of adult-generated DG GCL cells at the Long-Term time point, we quantified BrdU+ cells 3-mon post-irradiation. While at our Short-Term time point BrdU labels proliferating cells, at our Long-Term time point, those BrdU cells remaining reflect those cells or progeny that have survived until the 3-mon time point (Figure 1B). Supporting that the 3-mon post-injection period is sufficient for proliferating progenitors to mature into DG GCL cells [135], BrdU+ cells in the Long-Term group presented labeled nuclei reminiscent of mature DG GCL neurons, often with a punctate pattern (Figure 7A), as we have seen previously [41]. Quantitatively, 3-mon after ^12^C irradiation, there were 64% fewer BrdU+ cells in IRR versus Sham mice (Figure 7B), but a similar number of BrdU+ clusters between IRR and Sham mice (Figure 7C). Bregma analysis of BrdU+ cells revealed main effects of Treatment and Bregma, but no Bregma × Treatment interaction (Figure 7D). Post-hoc analyses of the 3-mon group revealed three bregma locations in the anterior DG where IRR mice had fewer BrdU+ cells relative to Sham mice. These data suggest decreased survival of DG GCL adult-generated cells in IRR mice relative to Sham mice, particularly in the anterior DG.

Another way to utilize the BrdU labeling design used for this experiment (Figure 1B) is to compare the percent of cells “surviving” between Short- and Long-Term groups in both Sham and IRR mice. This is useful since at the Short-Term time point there was already a significant decrease in the number of BrdU+ cells in IRR versus Sham mice (Figure 4B) [41]. To account for this pre-existing difference in BrdU+ proliferating cell number in the Short-Term groups, we calculated the percent decrease in BrdU+ cells across time points when comparing Sham to Sham and IRR to IRR (e.g., comparing Figure 4B to Figure 7B, calculations not shown). In Sham mice, there were 70% fewer BrdU+ cells in the Long-Term group relative to the Short-Term group. In IRR mice, there were 78% fewer BrdU+ cells in the Long-Term group relative to the Short-Term group. Thus, BrdU+ cells between the Short- and Long-Term time points have a similar survival rate in Sham and IRR mice.

### 2.7. Three-mon Post-Irradiation, IRR Mice Had Similar Number of DCX+ Cells Compared to Sham Mice

For the Long-Term group, DCX+ cell number was also quantified to gauge the number of immature neurons, as had been done in the Short-Term group. Qualitatively, DCX+ cells presented a similar range of morphologies in the Long-Term group (Figure 8A), as they did in the Short-Term group (Figure 5A). Quantitatively, Sham mice had 75% fewer DCX+ cells in the Long-Term group when compared to the Short-Term group (compare Figure 8B with Figure 5B, calculations not shown), indicative of the decrease in neurogenesis that happens with age [143,146]. In regard to effect of ^12^C irradiation, however, there was no difference in DCX+ cell number between Sham and IRR mice at the Long-Term time point (Figure 8B). Bregma analysis revealed a main effect of Bregma and a significant Bregma × Treatment interaction, but no main effect of Treatment (Figure 8C). Post-hoc analyses revealed one bregma position in the posterior DG with fewer DCX+ cells in IRR mice relative to Sham mice. Thus, at this Long-Term time point, the IRR mice had a similar number of DCX+ cells when compared to Sham mice, with fewer DCX+ cells only noted in the posterior DG.

### 2.8. 24-h and 3-mon Post-Irradiation, IRR Mice Had Similar GCL Volume and Similar Density of Immature Neuron Dendritic Processes Compared to Sham Mice

**Figure 9:**
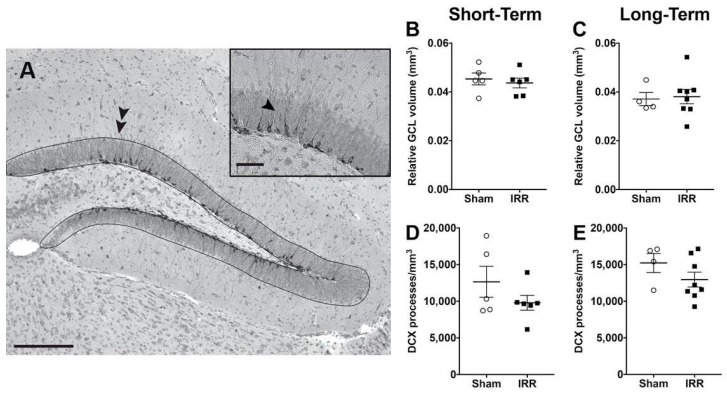
24-h and 3-mon post-^12^C irradiation, relative GCL volume, and the dendritic density of DCX+ cells were similar between Sham and IRR mice. (**A**) Main image: Representative photomicrograph of DCX staining depicting GCL boundary (dotted line, indicated by double arrowheads). When used in combination with the optical fractionator and Cavalieri’s principle, this approach reveals the relative GCL volume quantified in B and C. Inset: Higher magnification of the dendritic processes of DCX+ cells (arrowhead). The density of DCX+ dendritic processes perpendicular to the GCL is presented in D and E. Scale bar = 100 µm main image; 50 µm inset. (**B**,**C**) Relative GCL volume in Sham and IRR mice 24-h (Short-Term, B; *N* = 5 Sham, *N* = 6 IRR) and 3-mon (Long-Term, C; *N* = 4 Sham, *N* = 8 IRR) post-irradiation (*p*’s > 0.05). (**D**,**E**) Density of DCX+ dendritic processes perpendicular to the GCL in Sham and IRR mice 24-h (Short-Term, D; *N* = 5 Sham, *N* = 6 IRR) and 3-mon (Long-Term, E; *N* = 4 Sham, *N* = 8 IRR) post-irradiation (*p*’s > 0.05).

Changes in cell number may be accompanied by changes in volume of the underlying structure [147,148], even when the changes in cell number are new neurons in the DG GCL [149]. To assess whether the transient loss of adult-generated neurons we report here (Figure 3, Figure 4, Figure 5, Figure 6, Figure 7 and Figure 8) was accompanied by loss of GCL area and relative volume, we used unbiased stereology and Cavalieri’s principle (Figure 9A). Relative GCL volume at both the Short- and Long-Term timepoints was similar between IRR and Sham-treated mice (Figure 9B,C), suggesting no overt influence of the transient loss of new neurons.

Another index of GCL integrity—as well as of DCX+ neurons—is the number or density of perpendicular dendritic processes that extend from DCX+ neurons into the GCL and through the molecular layer [135,141,150,151,152]. Using each animal’s GCL cross-sectional area, we determined the density of these perpendicular DCX+ dendrites in IRR and Sham-treated mice at Short- and Long-Term time points (Figure 9A, inset). Similar to the result with GCL volume, the density of perpendicular DCX+ dendrites was similar between IRR and Sham-treated mice (Figure 9D,E).

## 3. Discussion

The goal of the present study was to define the effects of whole-body ^12^C irradiation on classic indices of neurogenesis in the young adult mouse DG GCL. Our results show that, relative to Sham mice, mice exposed to 100 cGy ^12^C irradiation (1000 MeV/n; 8 kEV/µm) have a transient decrease in the number of proliferating cells and immature neurons in the DG, but no change in new neuron survival rate. Specifically, at the Short-Term time point (24-h post-irradiation), there were fewer Ki67+ and BrdU+ cells and clusters (indicative of decreased proliferation) and fewer DCX+ cells (indicative of decreased immature neuron number) in IRR versus Sham mice. At the Long-Term time point (3-mon post-irradiation), the numbers of Ki67+ cells and clusters and of DCX+ cells were similar in IRR and Sham mice, suggesting the decrease in proliferation and immature neurons seen at the Short-Term time point was transient. This normalization of Ki67+ and DCX+ cell number underscores the regenerative capacity of the hippocampal DG several months after ^12^C irradiation. At the Long-Term time point, BrdU+ cell number remained lower in IRR vs Sham mice. While we did not double-label these 3-mon old BrdU+ cells with a neuronal marker, the vast majority of these surviving cells become neurons [41]. Thus, it might seem reasonable to conclude that ^12^C irradiation leads to a long-lasting decrease in DG neurogenesis. However, there are two reasons that we conclude there is a transient—and not a long-lasting—decrease in neurogenesis after ^12^C irradiation. First, there were fewer BrdU+ cells in IRR versus Sham mice at both the Short-Term and Long-Term time points, and there was a similar percent change (or survival rate) in BrdU+ cells from Short- to Long-Term within treatment. This suggests that the decreased BrdU+ cell number in IRR versus Sham mice at the Long-Term time point was due to the fewer proliferating BrdU+ cells at the Short-Term time point. Second, DCX+ cell number in IRR mice returned to the Sham level at the Long-Term time point, suggesting normalization of this marker of immature neurons by 3-mon after ^12^C irradiation. Taken together, these results show the ^12^C irradiation parameters used here result in a transient decrease in DG neurogenesis in the young adult mouse.

Our results with ^12^C irradiation and neurogenesis fit well with the prior publications examining the influence of other HZE particles (e.g., ^56^Fe, ^28^Si) on rodent DG neurogenesis. For example, smaller particles like ^12^C (studied here) and ^28^Si [41] tend to transiently decrease DG neurogenesis, while ^56^Fe leads to a more persistent decrease in neurogenesis [40,84]. However, our finding that ^12^C irradiation transiently decreases DG neurogenesis is in contrast with the only other ^12^C irradiation and DG neurogenesis publication [39]. There are several explanations for this apparent discrepancy in the influence of ^12^C irradiation on DG neurogenesis. First, the prior ^12^C irradiation work only examined one time point 9-mon post-irradiation and used sampling not stereology to quantify neurogenesis [39]. With the benefit of time, we were guided by the more recently-accepted view that stereology is a rigorous, accurate, and unbiased approach to measure neurogenesis [153,154]. Perhaps it was our use of stereological principles, as well as employing both Short- and Long-Term time points, which enabled our detection of the transient decrease in DG proliferating cells and immature neurons shown here. Second, the prior ^12^C irradiation work restricted analysis to the rostral and mid hippocampus [39]. This is notable since the hippocampus is an anatomically diverse and long structure, with distinct neural connections and functions along its longitudinal axis [45,155,156,157]. In fact, hippocampal function varies with distance from bregma: the anterior (also called dorsal or septal) hippocampus is linked to spatial learning and memory, while the posterior (also called ventral or temporal) hippocampus is linked to mood and emotions [4,42,45,158,159,160]. Neurogenesis, and neurogenesis-linked function, also varies along the longitudinal axis of the hippocampus [42,44,161,162,163,164,165]. Thus, we opted to assess ^12^C irradiation-induced changes throughout the whole hippocampal DG, rather than just the rostral and mid hippocampus. Interestingly, at the Short-Term time point in our study, there were fewer proliferating cells (Ki67+ and BrdU+ cells) and immature neurons (DCX+ cells) specifically in the posterior DG in IRR versus Sham mice. In contrast, at the Long-Term time point, there was a more mixed effect: no difference in cell proliferation (Ki67+ cells), fewer immature neurons (DCX+ cells) at one posterior bregma position, yet fewer surviving BrdU+ cells at several anterior bregma positions. While our experiment was not designed to assess behavioral changes in mice after irradiation, these bregma results and the large literature connecting neurogenesis across the longitudinal axis with distinct hippocampal functions indicate that future studies assessing mood-related behaviors soon after irradiation may be warranted.

Given that we find a transient decrease in neurogenesis in ^12^C irradiated mice when compared to Sham-treated mice, it is reasonable to consider whether there was an accompanying decrease in the volume of the GCL. However, GCL volume between IRR and Sham mice was similar at both the Short- and Long-Term time points. While we cannot exclude the possibility that GCL volume was transiently decreased at an unexamined timepoint, another index of GCL integrity—new neuron dendritic density—was also similar between IRR and Sham mice. The extension of a DCX+ dendritic process through the GCL enables its integration into the hippocampal trisynaptic network [135,141,150,151,152,166], and therefore it is an additional contributor to GCL volume. It is perhaps not surprising that ^12^C irradiation does not change DCX+ dendritic density since DCX+ cells with dendrites are post-mitotic, and HZE particles target proliferating cells rather than mature and migrating neuroblasts. Taken together, these data suggest the transient decrease in neurogenesis in ^12^C irradiated mice compared to Sham-treated mice was not accompanied by structural readjustments of the DG, and may be primarily due to an initial decrease in the number of proliferating cells.

It is also important to question the mechanism that contributes to or underlies the ^12^C irradiation-induced transient decrease in DG neurogenesis. Given the large literature on HZE particle irradiation and oxidative stress [26,71,88,93,113,114,167,168,169,170], a reasonable hypothesis is that oxidative stress and/or damage may play a role. Previously, we have reported indices of genomic instability in the DG GCL of mice seven days and two-mon post-^56^Fe IRR [40]. For the present study, we attempted to measure additional indices of oxidative stress after ^12^C irradiation using antigenic targets and IHC protocols from our lab and from the published literature [40,171,172,173,174,175,176,177]. However, we were unable to resolve any oxidative stress signal in tissue from the two time points studied. Since our data show that ^12^C irradiation transiently decreases neurogenesis but does not change the survival rate of new neurons between IRR and Sham mice, it is possible that oxidative stress signals are also transient, increasing after our Short-Term time point yet normalizing prior to our Long-Term time point. This hypothesis remains to be tested, ideally with alternative detection methods (e.g., western blotting of tissue samples rather than IHC), which may be sensitive enough to detect low levels of oxidative stress. If future experiments support this hypothesis, then therapies to combat oxidative stress may be best employed near the time of HZE exposure, as has been shown in preclinical studies [26,113,167,169].

While our current work with whole-body ^12^C irradiation has primary relevance to the ^12^C component of galactic cosmic radiation to which astronauts will be exposed during interplanetary travel, there is some clinical relevance as ^12^C has emerged as a focally-applied cancer treatment [34,35,178]. Previously, X-irradiation was utilized to treat cancers, but in order to achieve a therapeutic effect, the dose must be increased to a level that cannot be tolerated by surrounding tissue [179]. In contrast, ^12^C has a narrow Bragg peak, enabling high dose delivery to tumor cells, while maintaining acceptable doses to surrounding tissue [179,180]. Overall doses used in cancer are greater than the 100cGy ^12^C employed here, but patients do receive fractionated doses that are more in line with the dose given in this work [181]. Our finding that ^12^C irradiation transiently decreases DG neurogenesis in the young adult mouse brain may serve as a launch pad for answering questions that are relevant to radiotherapy as well as the influence of deep space travel on brain function.

## 4. Materials and Methods

### 4.1. Animals

For the experiments, (*n* = 24) male Nestin-CreER^T2^YFP/R26R-YFP (Nestin-CreER^T2^YFP) mice were used. As previously described [182], the mice were generated by breeding homozygous Nestin-CreER^T2^YFP mice on a C57BL/6J background with homozygous Rosa26Reporter:YFP knock-in mice to generate bitransgenic hemizygous for both genes. Mice had *ad libitum* access to food and water, were kept on a light/dark cycle of 12-h (lights on 6:00 AM), and were housed four per cage. At 5–7 weeks of age, Nestin-CreER^T2^YFP mice were injected with the estrogen ligand tamoxifen (TAM, i.p. 180 mg/kg/d, dissolved in 10% EtOH/90% sunflower oil, one injection/day for five consecutive days) [182]. Data from this inducible labeling (YFP+ cells) are the focus of a separate experiment, and are not shown in this current work. At 9–11 weeks of age, all mice were shipped to BNL in Brookhaven, New York and were allowed to acclimate for five days before irradiation (IRR) treatment (Figure 1A; “Irradiation Procedures” below). 22-h post-^12^C irradiation or Sham treatment, all mice received one injection of thymidine analog bromodeoxyuridine (BrdU; 150 mg/kg, i.p.; 10 mg/mL in 0.9% saline and 0.001M NaOH) to enable stereological assessment of stages of DG neurogenesis [183] with a dose of BrdU sufficient to pulse label all S-phase DG cells [129], as consistent with previous studies [40,41,84,184]. Half of the mice were killed 2-h post BrdU injection (24-h post-irradiation or Sham treatment) at BNL, and were named the “Short-Term” group (Figure 1A). The remaining “Long-Term” mice were shipped back to UT Southwestern Medical Center (UTSW), and killed 3-mon post-irradiation or Sham treatment (Figure 1A). Experimental protocols were approved by the Institutional Animal Care and Use Committee (IACUC, APN 0960-07-02-1) of both UTSW (approved April 2009) and BNL (approved October 2008), and mice were treated in accordance with National Institute of Health (NIH) guidelines.

### 4.2. Irradiation Procedures

Irradiation was carried out at BNL, similar to our prior work [40,41,84]. ^12^C particles were produced at the Alternating Gradient Synchrotron Booster at BNL and transferred to the NSRL facility experimental beam line [185], and the delivered beam was 20 cm × 20 cm (uniformity 5%). IRR mice were placed individually into ventilated and clean 50 mL conical tubes at a position perpendicular to the beam (heads positioned to beam center). IRR mice received whole-body exposure to 100 cGy ^12^C particles (1000 MeV/n, LET 8 KeV/μ) at a dose rate of 100 cGy/min. Control (Sham) mice were also shipped to BNL, transferred to NSRL, handled and placed in conical tubes for a similar amount of time as IRR mice, but were not exposed to the beam. The mice that are described in this work were run during the Spring 2010 NSRL campaign.

### 4.3. Tissue Preparation and Immunohistochemistry (IHC)

Either 24-h or 3-mon post-irradiation (Figure 1A), mice were sacrificed by live decapitation, and brains were subsequently extracted, bisected along the mid-sagittal suture, and post-fixed for <3 days prior to cryoprotection (30% sucrose in 0.2% sodium azide) at 4 °C. Brain hemispheres were sectioned coronally between bregma positions 0.02 and −4.84 (“bookends” to the DG) [122] at 30 µm in a 1:9 series and stored in 1× PBS with 0.01% sodium azide at 4 °C until processing for IHC.

To stain for neurogenesis relevant markers (Figure 1B) [183], slide-mounted IHC for DCX+, Ki67+, and BrdU+ cells in the DG was performed, as previously described [40,41,84,182]. One series of the hippocampus (every ninth section) was mounted onto Fisher Scientific Microscope Superfrost/Plus Precleaned charged slides from anterior to posterior and allowed to dry for 2 h. Antigen retrieval was performed using 0.01 M citric acid (pH 6.0) at 100 °C for 15 min, then was washed in PBS at room temperature. Endogenous peroxidase activity was inhibited by incubating with 0.3% hydrogen peroxide (H_2_O_2_) for 30 min. For BrdU IHC, two additional pretreatment steps were performed to allow the antibody access to DNA inside the cell nucleus: permeabilization and denaturation. Permeabilization was performed using 0.1% Trypsin in 0.1 M TRIS and 0.1% CaCl_2_, and denaturation was performed while using 2N HCl in 1× PBS. For all markers, non-specific binding was blocked with 3% serum (donkey) and 0.3% Triton-X in PBS for 1 h.

Following pretreatment and blocking, the slide-mounted sections were incubated with either rabbit-α-Ki67 (1:500; Fisher Scientific, catalog #RM-9106S, Freemont, CA, USA), rat-α-BrdU (1:500; Accurate, catalog #OBT 0030G, Westbury, NY, USA), or goat-α-DCX (1:8000; Santa Cruz Biotechnology, catalog #sc-8066, Dallas, TX, USA) in 3% serum and 0.3% Tween-20 overnight. After primary antibody, incubation with biotinylated secondary antibodies for DCX: biotin-donkey-α-goat-IgG (catalog #705-065-003), for Ki67: biotin-donkey-α-rabbit-IgG (catalog #711-065-152), or for BrdU: biotin-donkey-α-rat-IgG (catalog #712-065-153), all 1:200, all from Jackson ImmunoResearch, West Grove, PA, USA) for 1 h. Incubation with avidin-biotin complex was performed for 90 min (ABC Elite, Vector Laboratories, catalog #PK-6100). Immuno-labeled cells were visualized with metal-enhanced diaminobenzidine (DAB, Fisher Scientific, catalog #PI-34065, Pittsburgh, PA, USA) for 5–10 min. Finally, slides were incubated for four-min in the nuclear counterstain Fast Red (Vector Laboratories catalog #H3403). They were then dehydrated in increasing ethanol concentrations and Citrisolv, and cover slipped with DPX (Electron Microscopy Sciences, catalog #13512).

### 4.4. Stereological Cell Counts

Ki67+, BrdU+, and DCX+ DAB cells were quantified by two blinded observers while using stereological principles and an Olympus BX-51 microscope at 400× magnification, as described previously [130,186,187]. The slides were code prior to IHC, and the code was not broken until quantification of an individual experiment was complete. Consistency analyses were performed when comparing the two observers, whose counts were within 10% of each other. Immunoreactive cells were quantified in every 9th coronal hemisection in the granule cell layer (GCL) in the DG (for DCX, BrdU and Ki67) spanning the entire anterior-posterior axis of the hippocampus (−1.00 mm to −3.97 mm from Bregma). As the entire DG was examined via stereology, the number of sections per mouse varied per stereology principles [188,189,190]. Stereology was performed under bright field microscopy, and the total cell counts and clusters (Ki67 and BrdU) were multiplied by 18 to account for the whole DG: ×9 for every 9th section and ×2 because hemisections were stained and quantified. Data are presented as total GCL cell counts in all mice (Figure 3B, Figure 4B, Figure 5B, Figure 6B, Figure 7B and Figure 8B) and total cell clusters (Figure 3C, Figure 4C, Figure 6C and Figure 7C). Data are also presented along the longitudinal axis of the DG (distance from Bregma, Figure 3D, Figure 4D, Figure 5C, Figure 6D, Figure 7D, and Figure 8C).

### 4.5. Stereological Estimation of GCL Volume and Density of Immature Neuron Dendritic Processes

Unbiased stereological estimation of GCL volume was assessed using Cavalieri’s principle [191,192,193] via the optical fractionator approach with contour tracing in Stereo Investigator software (MBF Bioscience). In brief, using a Zeiss AxioImager M2 microscope at 200× magnification, an experimenter blind to treatment traced the boundary of the DG GCL in every 9th coronal hemisection to quantify the cross-sectional area. The sum of the areas of each animal was multiplied by the section thickness (30 µm) and converted to mm^3^, as previously described [194], to reveal the relative volume of the GCL for each animal.

Another index of GCL integrity that also provides insight into new neuron maturation is DCX+ dendritic analysis [135,141]. Young DCX+ cells have either no dendrite or a very short dendrite oriented nearly parallel to the GCL, while the older DCX+ cells have a dendrite oriented nearly perpendicular to the GCL, which branches and eventually projects into the DG molecular layer (e.g., Figure 1D, Figure 7A and Figure 9A). DCX+ cells of these different “ages” are differentially regulated, and quantification of their dendrites is an increasingly common subindex of neurogenesis [135,141,150,151,152]. Here, we focused on the older DCX+ cells by assessing the density of DCX+ dendrites oriented at 90° relative to the GCL. In brief, the sum of these specific DCX+ dendritic processes were quantified within each outlined GCL boundary and divided by the GCL area in order to reveal the density of processes from older DCX+ cells.

### 4.6. Statistical Analyses and Image Presentation

The data are displayed as mean ± SEM. Prism version 7.0 was used to perform the statistical analysis and *p* < 0.05 was defined as statistical significance. The statistics are reported in the Results section, in the figures (Figure 3, Figure 4, Figure 5, Figure 6, Figure 7, Figure 8 and Figure 9), and the figure legends. Total cell, cluster counts, GCL volume and dendritic process density (Figure 3B,C, Figure 4B,C, Figure 5B, Figure 6B,C, Figure 7B,C, Figure 8B and Figure 9B–E) were analyzed with two-tailed unpaired *t*-tests with the variable of Treatment: Sham versus IRR. Bregma analysis was done via two-way ANOVA Bregma x Treatment, and a post-hoc (Sidak’s multiple comparison) test was used to determine significance at specific bregma positions (Figure 3D, Figure 4D, Figure 5C, Figure 6D, Figure 7D and Figure 8C). Ki67+, BrdU+, and DCX+ cell and cluster survival rates were calculated as 100 × [(Short-Term cell/cluster count) − (Long-Term cell/cluster count)]/(Short-Term cell counts), similar to previous works [41,195]. Two mice were omitted following IHC due to tissue quality issues: one Sham mouse from the Short-Term DCX group and one IRR mouse from the Long-Term DCX group. Photomicrographs presented in this study were collected on an Olympus BX51 microscope with a DP74 camera with CellSens software or a Zeiss AxioImager M2 microscope while using a Lumina High Resolution Color Camera and StereoInvestigator software. Images were taken using 10, 20, and 40× objectives, and they were collected in full screen with manual exposure, gain 1×, Snapshot/Process: 1920 × 1200 (3COMS), and were exported as .jpeg or .tiff. Images were imported into Adobe Photoshop and saved, and a copy of the original image was subjected to the following: Image Size decreased while resolution increased to 300 dpi (with resampling), Mode changed to grayscale, Levels bounded to histogram with only gamma correction.

## Abbreviations

BNL	Brookhaven National Laboratories
BrdU	bromodeoxyuridine
CA1	cornus ammonis 1
CA3	cornus ammonis 3
cc	corpus callosum
DAB	diaminobenzidine
DCX	doublecortin
DG	dentate gyrus
GCL	granule cell layer
h	hour
HZE	high-energy and high-charge
IHC	immunohistochemistry
IRR	irradiated
Mol	molecular layer
mon	month
NSC	neural stem cell
NSRL	NASA Space Radiation Laboratory
or	stratum oriens
SEM	standard error of the mean
SGZ	subgranular zone
TH	thalamus
UTSW	University of Texas Southwestern Medical Center
